# Exploring the roles of trust and social group preference on the legitimacy of algorithmic decision-making vs. human decision-making for allocating COVID-19 vaccinations

**DOI:** 10.1007/s00146-022-01412-3

**Published:** 2022-04-21

**Authors:** Marco Lünich, Kimon Kieslich

**Affiliations:** Department of Social Sciences, Heinrich Heine University Düsseldorf, Düsseldorf, Germany

**Keywords:** Algorithmic decision-making, Trust, Social group preference, COVID-19, Decision legitimacy, Public opinion

## Abstract

In combating the ongoing global health threat of the COVID-19 pandemic, decision-makers have to take actions based on a multitude of relevant health data with severe potential consequences for the affected patients. Because of their presumed advantages in handling and analyzing vast amounts of data, computer systems of algorithmic decision-making (ADM) are implemented and substitute humans in decision-making processes. In this study, we focus on a specific application of ADM in contrast to human decision-making (HDM), namely the allocation of COVID-19 vaccines to the public. In particular, we elaborate on the role of trust and social group preference on the legitimacy of vaccine allocation. We conducted a survey with a 2 × 2 randomized factorial design among *n* = 1602 German respondents, in which we utilized distinct decision-making agents (HDM vs. ADM) and prioritization of a specific social group (teachers vs. prisoners) as design factors. Our findings show that general trust in ADM systems and preference for vaccination of a specific social group influence the legitimacy of vaccine allocation. However, contrary to our expectations, trust in the agent making the decision did not moderate the link between social group preference and legitimacy. Moreover, the effect was also not moderated by the type of decision-maker (human vs. algorithm). We conclude that trustworthy ADM systems must not necessarily lead to the legitimacy of ADM systems.

## Introduction

The global COVID-19 pandemic coincides with the ongoing worldwide proliferation of computer technology in everyday life. Consequently, computer systems have become widely regarded as a viable instrument with which to combat the pandemic (Bragazzi et al. 2020; Calandra and Favareto 2020; Jacob and Lawarée 2020; Malik et al. 2020; Nguyen et al. 2020; Sipior 2020). For instance, the medical research necessary to mitigate loss of life and to find treatment, cures, and vaccines for the virus is unthinkable without computers. Beyond their general use as research instruments for medicine and public health, computer systems are also helpful for mitigating pandemic-related social issues. As a prime example, algorithmic decision-making (ADM) systems have been used with the objective of automatically and fairly prioritizing persons for vaccination and to better coordinate the vaccination process. As vaccination prioritization is a hotly debated social issue and incautious use of technology may lead to severe social consequences, this implementation of ADM has received notable public scrutiny. In many cases in which ADM systems were deployed, it became quickly apparent that their prioritization results were biased, leading to backlash and outright rejection (Ciesielski et al. 2021; Guo and Hao 2020).

Nevertheless, even if ADM systems consistently followed a formally fair algorithm, as intended by their makers, the public might still question the algorithmic systems’ decisions despite their formal attainment of optimization goals. After all, algorithms may arrive at optimized decisions that correspond to formally correct and fair outcomes, but are unintuitive to a lay public, as decisions may entirely oppose social preferences and moral beliefs. Although negative evaluations of controversial public decision-making pose a general social problem, regardless of whether those decisions are based on human decision-making (HDM) or ADM, it is unclear if the type of decision-maker influences evaluation of that decision.

In this paper, to shed light on social issues related to adoption of this technology, we first ask to what extent ADM is perceived by the public as a viable solution for the distribution of the vaccine, and we examine the role of citizen trust as an explanatory factor in viability perceptions. Second, we investigate the impact of decisions related to prioritization of vaccine allocation on citizens’ perceptions of the legitimacy of the decision. In particular, we examine the consequences for perceived legitimacy when decisions are unpreferred by the public. Contrasting such perceptions concerning ADM to a situation in which humans make the prioritization decisions, we also examine whether citizens’ trust in the agent making the decision moderates the supposed relationship between the favorability of a decision and perceptions of its legitimacy, as well as whether the proposed mechanisms differ between the two decision-making agents. 

For the data representing attitudes among the German population, we draw a quota sample (criteria are gender, age, and educational level) from a German online access panel. Results indicate ambivalence in the general perception of ADM as a viable tool for disseminating COVID-19 vaccines among the German population. However, higher general trust in ADM systems is positively related to a favorable assessment of the viability of their use in vaccine distribution. Using a factorial survey design that randomly varies the vaccine prioritization of different social groups (prisoners versus teachers) and the agent deciding such prioritization (ADM versus HDM), results also suggest that decisions that assign a higher priority to unpreferred groups are perceived to be less legitimate. Contrary to the authors’ expectations, the trust in the agent making the decision did not moderate this relationship, and there is no difference between ADM and HDM concerning a moderating effect of trust.

As ADM systems may have adverse and especially discriminating consequences and the use of ADM systems hinges on widespread public acceptance, the resulting insights into the determinants of public support concerning ADM provide valuable information regarding their implementation. We consequently discuss implications for executives, politicians, and actors from civil society.

### Using ADM systems to prioritize COVID-19 vaccine distribution

The distribution of limited goods, such as medical resources and especially vaccines, is a social challenge that warrants research attention (Grover et al. 2020; Huseynov et al. 2020; Ratcliffe 2000). The prioritization of vaccination continues to be a hotly debated public issue as the world faces the threat of a global pandemic. The rollout of the international vaccination program against COVID-19 was complicated by a limited amount of the vaccine and the need to distribute it as rapidly and effectively as possible.

Such a vaccine distribution process often relies on many multi-faceted data points from patients, including their age, occupation, and pre-existing health issues, to determine individual risk status and make a decision about prioritization (World Health Organization 2012). The rule-based distribution then usually relies on technical formulations that structure and evaluate such input data according to pre-determined distribution criteria for the provision of vaccines.

The more data points that are considered and the more sophisticated the allocation formula, the more difficult it becomes for human decision-makers to establish an order for vaccination. Consequently, computer systems have been deployed to assist in this process (Ciesielski et al. 2021; Chiusi 2021; Guo and Hao 2020). Moreover, formalized algorithms and computer systems can be used to organize the pre-determined vaccination distribution, thus providing guidance in the identification and implementation of better optimized distributions. In a simulation study “using an age-stratified mathematical model paired with optimization algorithms” (Matrajt et al. 2021, 1), a research group shows how different optimizing strategies lead to different recommendations regarding vaccination prioritization.

In theory, if one aims for a fine-grained allocation based on extensive data-processing, digital tools may better optimize the allocation of vaccines and do so more quickly. Thus, an algorithm may also relieve medical or administrative staff in times of crisis. Consequently, ADM may be seen as a viable solution for allocating COVID-19 vaccines—at least when it comes to the bureaucratic perspective of public management and administrative decision-makers (Wirtz and Müller 2018).

In practice, despite great hopes for better outcomes, ADM systems have often not been able to protect what appear to be the most vulnerable groups and have led to unintended and morally questionable decisions. Deployed as a tool to prioritize people for vaccination against COVID-19, ADM systems, too, have shown to produce incorrect and biased decisions that have been regarded as morally wrong and unfair.^1^

In December 2020, when an algorithm was tasked with distributing the first batch of COVID-19 vaccines at the Stanford Medical Center in San Bernadino, California, only a few frontline physicians were prioritized (Guo and Hao 2020). While not all reasons for this result have been made public, a report by the *MIT Technology Review* highlights that the inclusion of the age of employees was critical, since the algorithm prioritized older staff members. However, according to the report, “frontline workers […] are typically in the middle of the age range” (Guo and Hao 2020). The report also notes that exposure to patients with COVID-19 was not included as a factor. The resulting algorithm’s preference for administrators or doctors working from home resulted in a backlash against the ADM system, protests from the hospital’s residents, and significant public attention (Wu and Isaac 2020).

In the German state of Bavaria in early 2021, an algorithm was used to assign vaccination appointments to a pre-defined risk group that consisted of people 80 years or older and younger persons with high-risk profiles, such as medical staff (Ciesielski et al. 2021). Appointments were prioritized for people with higher scores, and those scores were based on age. However, the algorithm assigned a randomly chosen value between 80 and 100 to persons below 80 years of age. Consequently, the algorithm discriminated against the younger octogenarians, who were simply assigned their true age and thus were assigned a lower priority than were some of the younger people. The chance of being prioritized as if they were older than 80 years is 95% for the younger persons in the risk group. As a result, only a few 80-year-olds received an appointment for vaccination, causing complaints and extra effort and expenses, as the underrepresented group had to be manually contacted.

## Theory, research questions, and hypotheses

### ADM for the common good and its public perception

Such anecdotal evidence is in line with recent research which suggests that the implementation of ADM in public administration has been far from smooth (Hartmann and Wenzelburger 2021). Even the most well-intentioned ADM may falsely discriminate against certain groups; such systems often violate the “established weighting of relevant ethical concerns in a given context” (Heinrichs 2021, 1). These general concerns regarding discrimination and biases have recently instigated substantial research activity that addresses the social implications of ADM implementation (Crawford et al. 2016).

To better guide the intricate development process of automated computer systems for the Common Good, Berendt (2019, 44) proposes four questions that must be asked regarding the means and end of ADM implementation: “What is the problem […]?” “Who defines the problem?” “What is the role of knowledge?” and “What are important side effects and dynamics?” Berendt points out that the Common Good is not clearly defined in the research community and might relate to criteria of fairness, accountability, transparency, or advocacy for those who are disadvantaged. However, Berendt stresses that one joint goal that should be pursued is making AI for the Common Good accessible to as many people as possible (and not only those who invest in the technology). Accordingly, fighting the pandemic threat by distributing vaccines, a goal that certainly should be beneficial to all, can be addressed through use of ADM. However, any attempt at appropriately answering Berendt’s questions reveals that implementing ADM in this situation may prove a complex and intricate task. Depending on different assumptions and preferences, the approaches to and results of ADM may vary extensively. For example, the respective solutions to the problem of too many infections or deaths, or too much economic damage, could be defined as either “lower case numbers (of certain groups),” “lower the death rate,” or even “ensure a fast return to normal life,” or all of the above. Furthermore, the problem might be defined by various stakeholders, e.g., experts, politicians, the media, or the general public. Additionally, one must consider how the problems and solutions that utilize ADM are framed by stakeholders via mediated public communication and how they are received and understood by all parties involved, especially the public. Eventually, it is difficult to determine ADM’s important side effects and unintended dynamics well in advance of utilization.

Consequently, to better guide the implementation process and prevent problems regarding the Common Good, the European Union offers specific guidelines that include promoting trustworthy ADM as a solution for opaque and inaccessible applications. However, setting up guidelines does not guarantee the development and implementation of AI for the Common Good. Other influences, like economic competitiveness or the satisfaction of specific stakeholder groups, might even stand in opposition to this goal (Hagendorff 2020). Therefore, analyzing public perceptions of ADM systems becomes even more relevant, since the public can articulate demands and put pressure on those in charge of developing and implementing such systems. In other words, all decisions concerning societal implementations of AI need to be legitimate. Regarding the concept of legitimacy, based on a literature review, Arnesen (2017) notes:

Actions are facilitated when the affected individuals comply. Compliance can be achieved through the use of various forms of power, such as money, social status or the use of force. Another way of facilitating action occurs when the affected individuals expect and consent to the action taking place. The term “legitimacy” conveys this concept of wilful [sic] compliance toward an action. (p. 148).

The role of trust, as it is highlighted by the EU Commission, is especially relevant in this context, as people who put trust in an entity are presumably more eager to legitimize respective decisions (Wiencierz and Lünich 2020). We note that we cannot, in this paper, judge the trustworthiness of an ADM system for vaccine allocation in a technical sense, and one might argue that strategic communication can lead to the perception of an ADM system as being trustworthy when it is not. Regardless, we are interested in the perception of trust in ADM for vaccine allocation, since trusting ADM systems, no matter if that trust is deserved, may affect perceptions of legitimacy. With that in mind, the use of ADM to combat the COVID-19 pandemic raises important research questions that we investigate in this study by adopting a focus on public perceptions of ADM discrimination and trust in the decision-making agent.

As a result, our paper contributes to the pre-existing literature in three important ways. First, it provides novel insights into the public perception of the use of ADM to combat the spread of COVID-19. Second, it sheds light on potential issues related to ADM implementation, primarily when the resulting decisions are perceived as unpreferable. Third, it addresses the effect of trust in the agents making important decisions, specifically regarding human and algorithmic decision-making.

### The perceived viability of ADM in the distribution of vaccines

To address the consequences of implementing ADM systems in this context, we must first examine the general public perceptions and assessments of the viability of ADM in the vaccine distribution process. In general, expectations concerning the use of ADM systems in decision-making include that the decisions will be quicker, more consistent, and in general more robust than human decisions when adhering to specific distribution formulas (Dawes et al. 1989; Kaufmann and Wittmann 2016; Kuncel et al. 2013). Accordingly, the use of ADM systems in public administration has gained considerable traction in recent years (Wirtz and Müller 2018) and—as demonstrated by the two case examples from the US and Germany—has also been implemented for the distribution of COVID-19 vaccines.

In general, people may be aware of the possibilities provided by computer systems and their implementation regarding distribution processes, even if they have not yet heard of specific ADM use cases for vaccine distribution or to achieve other goals. For instance, there is a general awareness concerning the widely discussed impact of Artificial Intelligence (AI) on society (Kelley et al. 2019). Strictly speaking, ADM may not necessarily be AI; however, in terms of public perception, it still can be argued that computer systems that autonomously make decisions are at least associated with AI from a lay perspective (Cave et al. 2019; Liang and Lee 2017). Research shows that citizens of many countries have rather favorable attitudes toward AI (Kelley et al. 2019; Zhang and Dafoe 2019). However, the consequential decisions of AI may be perceived as threatening in certain contexts, such job recruitment, and loan origination (Kieslich et al. 2021b).

Further studies report that trust in algorithms “depends on the characteristics of the task” (Castelo et al. 2019, 26). Trust was generally found to be high when algorithms perform tasks that are perceived as being computational, do not involve emotional competencies, and have outcomes with limited consequences. On the other hand, Araujo et al. (2020) found that ADM systems were perceived as more useful in high-impact situations, i.e., in situations that may have serious consequences.

Given the mixed evidence of previous studies, we crafted our first research question around the perceived viability of ADM in the distribution of vaccines.

*RQ1. To what extent do people consider ADM a viable solution for the distribution of COVID-19 vaccines?*

### Trust in algorithms and the perceived viability of ADM

ADM systems are often considered “black boxes”, because it is usually impossible to make the inner workings of such systems transparent and comprehensible (Ananny and Crawford 2018). They operate with millions of data points and predict outcomes using opaque self-learning algorithms. Most systems are so complex that even developers and researchers sometimes fail to understand how the machine came to a specific conclusion (Burrell 2016; Diakopoulos 2016). The high complexity of such systems may lead to a lack of comprehension, especially among those in the general public with little technical knowledge about the underlying technology (de Fine Licht and de Fine Licht 2020). Consequently, a comprehensive understanding of the system’s data-driven decisions can be difficult to achieve.

In such situations, trust becomes an essential and influential factor in the formation of attitudes and in decision-making. This study adopts a prominent definition of trust that has also been adopted by AI researchers (e.g., Glikson and Woolley 2020):

the willingness of a party to be vulnerable to the actions of another party based on the expectation that the other will perform a particular action important to the trustor, irrespective of the ability to monitor or control that other party. (Mayer et al. 1995, p. 712).

Thus, due to the complexity of the systems, people are often put in situations where they have to rely on the decisions of ADM without being able to personally check and verify whether the decision-making process and the final result are acceptable.

Hence, a common goal of researchers and politicians is to create systems that are trustworthy. For instance, the European approach to AI has been crafted by a high-level expert group that actively strive for trustworthy AI design, and this goal also applies to ADM systems (European Commission 2019). According to the EU guidelines, trustworthiness can be achieved through the fulfillment of seven ethical principles and resulting key requirements for intelligent systems: human oversight, technical robustness and safety, privacy, transparency, fairness, societal and environmental well-being, and accountability (for an overview of global ethical guidelines, see Jobin et al. 2019). The main intention of the guidelines is to strengthen public trust in such systems, which will subsequently lead to acceptance of their implementation.^2^

**Fig. 1:**
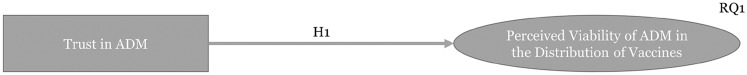
The conceptual model for the RQ1 and H1

Empirical research shows that trust is a driver of positive opinions about technology acceptance (Hoff and Bashir 2015). Shin (2021b) and Shin (2021d) found evidence that trust in algorithms positively influences perceptions of algorithmic performance. Moreover, Shin (2021a) showed that, in the case of an evaluation of a chatbot, algorithmic trust differences among participants (lower trust versus higher trust) were associated with varying levels of credibility assessment and different information-seeking behavior. Positive evaluations of credibility increased alongside levels of algorithmic literacy and trust in algorithms. Additionally, Shin and Park (2019) reported that people who show high trust in algorithms evaluate algorithms more positively in terms of satisfaction and usefulness than do those who show less trust in algorithms. In another study that focused on the evaluation of algorithmic recommendation applications, Shin (2020b) confirmed the positive link between trust and perceptions of usefulness and found that higher trust levels are also associated with higher levels of perceived convenience. Furthermore, Shin (2021d) showed that trust had a mediating role on the emotional reaction to algorithmic recommendations for the independent variables of perceptions of algorithmic transparency, fairness, accountability, and explainability. Shin (2021c) additionally showed that, after a chatbot gave recommendations to participants, perceived algorithmic trust had a positive effect on both the performance rating of algorithmic accuracy and the perceived quality of personalization. Experimental research by Robinette et al. (2016) further suggested that participants followed algorithmic instructions given by a robot in a high-risk situation due to (over)trust, even after seeing it make mistakes. Accordingly, we hypothesize as follows (see Fig. 1):

*H1. Trust in ADM will be positively related to acceptance of ADM as a viable solution for distribution of COVID-19 vaccines*.

### Social preferences in the evaluation of the distribution process

The general assessment of ADM’s viability, however, is only a tiny piece of the social puzzle of vaccine distribution. A more significant issue is related to the actual decision-making and its results. Just because people perceive a decision-making as viable in general, a resulting decision itself is still individually evaluated and may subsequently be questioned due to various reasons.

After all, even if an ADM system consistently arrives at formally fair decisions, this does not automatically mean that the decisions will be widely endorsed. In this regard, ADM decisions are similar to decisions made by humans. For instance, people may still call into question the algorithmic systems’ decisions despite formal attainment of optimization goals, whether because those decisions are unintuitive, incomprehensible, or in opposition to an individual’s social preferences and moral ideas (Brown et al. 2019; Grgic-Hlaca et al. 2018).

When it comes to the actual distribution of limited public goods (e.g., the distribution of vaccines against COVID-19), decisions that favor one social group over another may thus prove to be a problem. The literature on the assessment of distribution problems has repeatedly shown that people exhibit not only material self-interest in their evaluation of decisions but also social preferences. “A person exhibits social preferences if the person not only cares about the material resources allocated to her but also cares about the material resources allocated to relevant reference agents” (Fehr and Fischbacher 2002, C2).

Ultimately, when applying the questions by Berendt (2019) mentioned above to the allocation of vaccinations, it is likely that different results may occur; hence, different ADM systems can be developed and deployed. If one aims to identify the persons at highest risk of catching COVID-19, particularly vulnerable groups are prisoners and teachers (Burki 2020; Gaffney et al. 2020; Kahn et al. 2020). Thus, an ADM system may derive a solution that treats both groups equally or may prioritize one group over the other.

However, it has been demonstrated by prior research (Fallucchi et al. 2021; McKneally and Sade 2003) that such decisions about the allocation of medical resources via the prioritization of different social groups will be questioned and perceived as illegitimate, as people regard them as objectionable on moral grounds. For instance, several studies found that patients’ characteristics and lifestyles influenced public perception regarding who should receive priority for organ transplantation. People would allocate significantly less medical treatment to smokers, to persons with high alcohol consumption, and to those who exhibit promiscuous behavior (Furnham et al. 2007; Huynh et al. 2020; Ubel et al. 2001). Personal life choices can lead to a preference for one affected group over another in the eyes of the public.

Consequently, the potential decision outcomes of ADM applications must be clarified, especially those that may be perceived as controversial. In this study, we do not wish to disentangle the specific motivations for a social preference in a particular decision. Our investigation instead focuses on the consequences of decisions that violate social preferences. Despite the best intentions, ADM, as well as HDM, may frequently result in controversial and unpopular decisions.

Concerning the allocation of scarce medical resources during the COVID-19 pandemic, studies have shown that the public prioritized treatment of younger patients and those that were comparatively sickest (Grover et al. 2020; Huseynov et al. 2020). Applied to the context of our study: Especially when public sentiment suggests that unfavored groups should not be receiving any advantages, decisions regarding vaccine distribution that are perceived as unfavorable may also be considered illegitimate by the public. For instance, prisoners are being punished for a crime and are subsequently often stigmatized and disadvantaged (Falk et al. 2009; Kjelsberg et al. 2007), especially in contrast to teachers, who are highly respected by the majority of the German population (dbb beamtenbund und tarifunion 2020). Decisions that favor a group with low social prestige over a group with high social prestige may be publicly questioned, as the need and merits of the latter group are considered more significant than those of the former group—irrespective of the algorithmic conclusions aimed at optimization that drove the decision-making in the first place. Therefore, it is assumed that in cases where decisions favor groups that are of lower social prestige, the disapproval of early vaccination of that group by the general public will result in lower perceived legitimacy of that decision. Accordingly, we hypothesize as follows:

*H2. The disapproval of early vaccination of a social group will be negatively related to the legitimacy of early vaccination of that group*.

### The moderating role of trust in the agent making the decision

Concerning the importance of trust in the evaluation of ADM discussed above, it may not only be an explanatory variable when it comes to general perceptions of the viability of ADM applications. Trust may more specifically be a decisive factor in a situation in which people encounter a decision by an agent that (a) is not fully comprehensible to them or (b) results in an outcome that they consider objectionable. Trust may then be a deciding factor, as people that show higher trust may still perceive a decision as legitimate even though they do not prefer the outcome. People with lower trust in the agent making the decision will perceive the decision as illegitimate.

Empirical research has shown that trust in algorithms moderates the effects of transparency, fairness, and accountability perceptions on satisfaction with an algorithm; for people with a high trust level, the positive effect of the relationship between the perception of and satisfaction with the ethical principles was higher than for those with low trust (Shin and Park 2019). Another study by Ye et al. (2019) focused on utilizing AI in medicine in China and found that trust in AI and medical staff negatively moderated the effect of the perceived usefulness on intention to use the respective technology. Hence, we hypothesize as follows:

*H3. Trust in the agent making the decision will moderate the negative relationship between disapproval of early vaccination of a social group and the legitimacy of early vaccination, such that this negative relationship will be weaker when trust in the agent making the decision is higher.*

### Differences between automated decision-making and human decision-making

The general reason for implementing ADM systems is to arrive at better decisions than those produced by human decision-making (König and Wenzelburger 2021). For instance, the use of ADM in public administration is often expected to be superior, both faster and cheaper as well as more reliable, impartial, and objective than HDM (Wirtz and Müller 2018). However, even if that were the case, the public assessment of important decisions may deviate for the various reasons suggested above. Despite the best intentions, decisions by both ADM and HDM, while technically correct and optimized for the desired results, may still be negatively perceived.

In this regard, two contrasting strands of the literature highlight the acceptance or rejection of algorithms and algorithmic advice, respectively, compared to human judgment: algorithmic aversion (Dietvorst et al. 2015; Dietvorst and Bharti 2020; for an overview, see Burton et al. 2020) and algorithmic appreciation (Logg et al. 2019). Notably, these studies focus on algorithms that cannot be perfect in their predictions, which always come with some degree of uncertainty. Such algorithms are used for daily recommendations or forecasting tasks.

Algorithmic aversion studies mostly argue that humans are preferred to algorithms, even if the latter perform better. Seminal work was done by Dietvorst et al. (2015), who found empirical evidence that people reject algorithms when they have seen them make a mistake. This finding persisted when participants directly compared an algorithm that made factually better decisions than those of a human. In another study, Dietvorst and Bharti (2020) argue that algorithmic aversion is correlated with the uncertainty of a given situation. If a problem cannot be solved deterministically but can only be derived by a system (e.g., the prediction of stock market prices), the higher the uncertainty of a situation, the more algorithms are rejected. Adding to this, Wojcieszak et al. (2021) showed that AI in online moderation was generally evaluated more negatively than was human moderation, which may be traced back to the unfamiliarity people have with algorithmic moderation.

On the other hand, Logg et al. (2019) found that laypeople more strictly followed the advice of algorithms than they did that of non-expert humans. However, this algorithm appreciation vanished when a human expert gave advice or when participants were forced to choose between their personal prediction and an algorithmic one. These findings are supported by Thurman et al. (2019), who tested algorithmic appreciation in news recommendations and found that algorithmic recommendations were preferred to expert recommendations. This empirical evidence was partly replicated by Wojcieszak et al. (2021), who reported that, in Spain, AI news recommendations were perceived as more favorable than news recommendations by journalists or editors; however, both kinds of recommendations were given equal weight in Poland and the USA.

Thus, several factors seem to play a role in the acceptance or rejection of algorithms, especially if a comparison is drawn to human decision-making. First, the context in which an algorithm is used is important. Studies suggest that uncertainty about a situation can lead to different degrees of algorithmic acceptance. Second, the level of expertise of the human to which the algorithm is compared plays a crucial role. If human decision-makers or advisers are considered experts, they are mostly preferred over algorithms, even if the results of their decisions are objectively worse. However, this is not true in all contexts.

In our study, we argue that if an ADM system makes a decision on vaccine distribution, the negative effect of trust will be weaker than it would be for human decision-making. This is because we consider vaccine distribution to be a high-risk situation, and former studies have shown that people rely on ADM and evaluate it more positively in those situations. For example, Araujo et al. (2020) showed that ADM systems were evaluated as more useful and fair and as having lower risks than human expert judgment in a scenario of high-impact decision-making. In a similar vein, Marcinkowski et al. (2020) found that ADM was perceived as fairer than HDM in a high-impact education use case. Furthermore, Robinette et al. (2016) reported that people relied on algorithmic advice in high-stakes situations, even if that advice was nonsensical. Concerning algorithmic recommendation, Logg et al. (2019) found that people rely more on machine advice than on human advice in a multitude of contexts. Accordingly, we hypothesize:

*H4. The type of agent making the decision moderates the interaction effect of the disapproval of vaccination of a social group and trust in the agent making the decision on the legitimacy of the decision, such that the negative relationship between disapproval of vaccination and perceived legitimacy of early vaccination is weaker when ADM makes the decision when trust in ADM is high than when humans make the decision when trust in humans is high*.

**Fig. 2:**
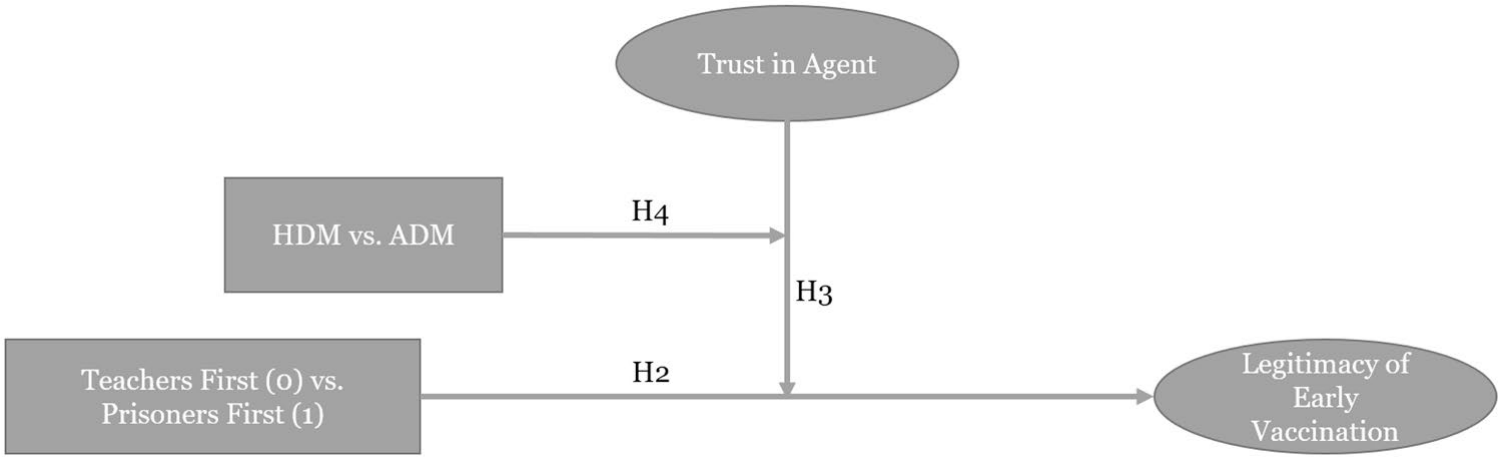
Conceptual model for H2–H4

Figure 2 shows the conceptual model for Hypotheses 2, 3, and 4.

## Method

To answer the research question and hypotheses, we conducted a cross-sectional factorial survey using an online questionnaire with standardized response options. The online survey facilitates a fast and cost-effective implementation of the research project, especially against the background of contact avoidance during the pandemic. A standardized survey also enables statistical tests of the research questions and hypotheses for the population of interest. Finally, the factorial survey design allows for the necessary comparison of the distinct approaches to allocation of the COVID-19 vaccines (using either ADM or HDM) as well as contrasting reactions to different allocation results.

To assess the findings, we performed the data analysis in R (version 4.0.3) using the packages *lavaan* (Rosseel 2012) and *semTools* (Jorgensen et al. 2019). R and both additional software packages used for data analysis are free and open source. We pre-registered our research question, hypotheses, and measurement of the variables (https://osf.io/xhvwr).

### Procedure and survey design

For screening purposes, respondents first had to provide some demographic information. Afterward, they answered questions concerning their opinions on the current COVID-19 pandemic, specifically on the political handling of the situation and their opinions on the current state and progress of vaccination. We also included questions about hypothetical vaccination prioritization of different social groups and trust in the standing commission on vaccination (STIKO), which is in charge of recommending vaccine prioritization in Germany. Next, after assessing subjective knowledge of artificial intelligence (AI),^3^ participants were given a brief explanation of the term AI, and they subsequently answered questions regarding their attitudes and opinions on it. Participants were then introduced to the use case—vaccination distribution through an ADM system. Thereby, ADM was explained as a form of AI. Respondents rated their trust in such a system before they were confronted with the experimental condition.

Each participant, following a 2 × 2 design, was presented with one of four possible scenarios. Participants were told that *either* an ADM system *or* the STIKO (a human commission/HDM) set up a vaccination distribution plan with the result that *either* teachers *or* prisoners would be prioritized. Respondents rated the legitimacy of the decision as well as their fairness perception of the distribution process. Finally, participants were thanked, debriefed, and redirected to the provider of the online access panel (OAP), where they received monetary compensation for participation.

### Sample

Participants were recruited via the online access panel (OAP) of the market research institute respondi, which is certified according to ISO 26362. To avoid overrepresentation and skew in the sample composition, quotas for gender, age, and educational level were used as a stopping rule. For example, once enough respondents of a certain age participated, no new participants from that age group were able to do so.^4^ Survey field time was between March 26 and April 12, 2021. At that time, vaccination against COVID-19 was not available to the general public in Germany; instead, it was dependent on pre-defined risk groups identified by the STIKO.

A total of 12,000 respondents from the OAP were invited to participate in the survey. The questionnaire was accessed by 3359 persons, of which 3,048 shared their socio-demographic data. Of those, 1184 were screened out, because their respective quotas were already exhausted or because they did not belong to the investigated population and were therefore ineligible for our survey. In total, 1740 respondents completed the questionnaire successfully. The dropout rate was 6.1%, and dropouts were equally distributed over all pages of the questionnaire. We filtered out those participants who answered the questionnaire in less than 4 min and 30 s (in a pre-test, the authors determined this to be the minimum amount of time needed to reasonably answer the questionnaire), which resulted in an exclusion of 138 datasets (7.93%). The final sample consists of 1602 participants.

The average age was 48.02 years (*SD* = 15.17). Altogether, 814 (50.8%) respondents identified as women and 788 (49.2%) as men. Furthermore, 512 (32.0%) respondents reported basic educational attainment, 536 (33.5%) reported medium educational attainment, and 554 (34.6%) hold an advanced degree.

### Measurement

*Approval of early vaccination for a social group* For the measurement of preference for early vaccination of a social group, respondents were asked, “How would you feel if the following groups of people were given priority in the distribution of the vaccine?” Afterward, they were presented with a self-developed list of social groups that included both teachers and prisoners in the closed penal system.^5^ Respondents were asked to rate on a five-point Likert scale (1 = do not like; 5 = like; − 1 = cannot judge) how they would feel if each group received prioritization for an early vaccination against COVID-19. A Welch’s t test shows that an early vaccination of teachers (*M* = 4.31, *SD* = 1.04) was significantly more preferred than an early vaccination of prisoners in the closed penal system (*M* = 2.09, *SD* = 1.30, *t*(2549.8) = − 49.83, *p* < 0.01).

*General trust in ADM* The general trust in ADM was measured via four items on a five-point Likert scale ranging from 1 = do not agree at all to 5 = totally agree. While the underlying construct is called *general trust in ADM*, the wording addressed systems of AI. We used this approach, because (a) we assumed a greater familiarity with the term *artificial intelligence* than with *algorithmic decision-making* and (b) the tested scales used for the assessment of our constructs were adopted from similar research contexts that predominantly referred to AI. The scale was adapted from the measurement of trust in recommender AI proposed by Shin (2021b), and the four items read as follows:

- “I trust that AI systems can make correct decisions.” (Variable ID: VT08_01).
- “I trust the decisions made by AI systems.” (Variable ID: VT08_02).
- “Decisions made by AI systems are trustworthy.” (Variable ID: VT08_03).
- “I believe that decisions made by AI systems are reliable.” (Variable ID: VT08_04).

The four indicators suggest good factorial validity (see Table 3).

*Viability of ADM for vaccine distribution* Assessing the perceived viability of ADM for vaccine distribution involved respondents rating three statements on a five-point Likert scale ranging from 1 = do not agree at all to 5 = totally agree. These three statements are as follows:

- “Computer-based decision systems are useful for the vaccine distribution process.” (Variable ID: AK04_01).
- “I support the use of computer-based decision systems in the vaccine distribution process.” (Variable ID: AK04_02).
- “The use of computer-based decision systems for vaccine distribution would help solve the problems of vaccine distribution.” (Variable ID: AK04_03)

The three indicators suggest good factorial validity (see Table 3).

*Trust in the agent (ADM/HDM) making decisions about vaccine distribution* Trust in ADM for vaccine distribution was measured in the same way as was general trust in ADM, except that we changed the word “AI” either to “a computer system in the vaccine distribution,” or to “the STIKO in the vaccine distribution”, respectively.^6^

Before assessing group differences using latent factor modeling, the necessary measurement invariance of the indicators (Putnick and Bornstein 2016) was examined through the following procedure. The first model assessed configural invariance (M1). In the second model (M2), we checked for metric invariance by constraining the factor loadings and comparing the two models using a ${\chi }^{2}$ difference test and assessing the difference of the TLI. A non-significant ${\chi }^{2}$ difference test suggests that the model with equality constraints does not fit worse than the model without such constraints, and the respective model parameters are considered to be equal. Afterward, a third model (M3) with constrained indicator intercepts was used to check for scalar invariance by comparing it to M2 using a ${\chi }^{2}$ difference test and assessing the difference of the TLI. A model that passes this test for measurement invariance suggests strong factorial invariance. The final step included constraining the residual variances of the indicators in a fourth model (M4), which we tested for residual invariance.

Table 1 suggests that there is factorial invariance for the measurement of trust in the agent making decisions regarding vaccine distribution. The four indicators suggest good factorial validity (see Table 3).

**Table 1:** Measurement invariance trust

	*χ*^2^ (*df*)	TLI	RMSEA (90% CI)	Model comp	Δ *χ*^2^ (Δ*df*)	ΔTLI	ΔRMSEA
M1: configural invariance	10.73* (4)	1.00	0.05 (0.01–0.08)				
M2: metric invariance	13.76 (7)	1.00	0.03 (0.00–0.06)	M1	3.03 (3)	0.00	− 0.02
M3: scalar invariance	18.94* (10)	1.00	0.03 (0.01–0.06)	M2	5.18 (3)	0.00	0.00
M4: residual invariance	31.26* (14)	1.00	0.04 (0.02–0.06)	M3	12.33* (4)	0.00	0.01

*p < 0.05

^a^*N* = 1602

*Legitimacy of the decision *regarding* vaccination prioritization* Legitimacy of the decision was measured with four items on a five-point Likert scale ranging from 1 = do not agree at all to 5 = totally agree. The scale items were adopted from Starke and Lünich (2020) and read as follows:

- “I accept the decision.” (Variable ID: LG04_01).
- “I agree with the decision.” (Variable ID: LG04_02).
- “I am satisfied with the decision.” (Variable ID: LG04_03)
- “I recognize the decision.” (Variable ID: LG04_04).

A test for measurement invariance suggests factorial invariance of the indicators measuring the legitimacy of the decision (see Table 2). The four indicators suggest good factorial validity (see Table 3).

**Table 2:** Measurement invariance legitimacy

	*χ*^2^ (*df*)	TLI	RMSEA (90% CI)	Model comp	Δ *χ*^2^ (Δ*df*)	ΔTLI	ΔRMSEA
M1: configural invariance	134.23* (8)	0.94	0.20 (0.17–0.23)				
M2: metric invariance	146.93* (17)	0.97	0.14 (0.12–0.16)	M1	12.71 (9)	0.03	− 0.06
M3: scalar invariance	157.65*(26)	0.98	0.11 (0.10–0.13)	M2	10.72 (9)	0.01	− 0.03
M4: residual invariance	214.38* (38)	0.98	0.11 (0.09–0.12)	M3	56.73* (12)	0.00	0.00

*p < 0.05

^a^*N* = 1602

**Table 3:** Reliability values

	Trust in ADM	ADM as viable solution	Trust in agent (ADM)	Trust in agent (human)	Legitimacy
alpha	0.95	0.94	0.97	0.97	0.95
omega	0.95	0.94	0.97	0.97	0.95
omega2	0.95	0.94	0.97	0.97	0.95
omega3	0.95	0.94	0.97	0.97	0.95
avevar	0.84	0.85	0.90	0.90	0.82

Accordingly (due to factorial invariance), when using the latent factors of *trust in the agent making the decision* and *legitimacy of the decision regarding vaccination prioritization* in the structural regression models of the analysis, equality constraints between the groups were imposed on the factor loadings, indicator intercepts, and residuals.

## Results

*Viability of ADM for vaccine distribution* To address RQ1, we ran a latent factor analysis. In this analysis and subsequent steps, effect coding was used for factor scaling, a procedure that “constrains the set of indicator intercepts to sum to zero for each construct and the set of loadings for a given construct to average 1.0” (Little et al. 2006, 62). The eventual factor is scaled like the indicators; especially in the case at hand, this helps with interpretation. As there were three indicators, the model is fully identified and there are no degrees of freedom and no model fit (see Table 4).^7^

**Table 4:** Latent factor model for the perceived viability of ADM for vaccine distribution

	Estimate (Std.Err.)	*p*
	*Factor loadings*	
Viability of ADM		
AK04.01	1.01(0.01)	0.000
AK04.02	1.02(0.01)	0.000
AK04.03	0.97(0.01)	0.000
	*Intercepts*	
AK04.01	0.06(0.03)	0.044
AK04.02	– 0.11(0.03)	0.000
AK04.03	0.06(0.03)	0.063
	*Latent intercepts*	
Viability of ADM	2.88(0.03)	0.000
	*Latent variances*	
Viability of ADM	1.29(0.05)	0.000
	*Fit indices*	
*χ*^2^	0.00(0)	
CFI	1.00	
TLI	1.00	
RMSEA	0.00	

Given the measurement on a five-point Likert scale, the mean of the latent factor (*M* = 2.88, *SD* = 1.14, *CI 95*(2.82; 2.94)) suggests that on average, the respondents were undecided regarding whether ADM is a viable solution for the distribution of the vaccine (RQ1). All in all, there was no outright endorsement or rejection of ADM systems for vaccine distribution.

*Relationship between general trust in ADM systems and the viability of ADM for vaccine distribution.* To test the hypothesis of a positive relationship between the general trust in ADM and the viability of ADM for vaccine distribution (H1), a structural regression model was utilized that included both constructs as latent factors. The model shows good fit^8^ (see Table 5).

**Table 5:** Structural regression model for the relationship between general trust in ADM and the viability of ADM for vaccine distribution

	Estimate (Std.Err.)	*p*
	*Factor loadings*	
Viability of ADM		
AK04.01	1.00(0.01)	0.000
AK04.02	1.02(0.01)	0.000
AK04.03	0.97(0.01)	0.000
General Trust in ADM		
VT08.01	1.03(0.01)	0.000
VT08.02	1.00(0.01)	0.000
VT08.03	0.97(0.01)	0.000
VT08.04	1.00(0.01)	0.000
	*Regression slopes*	
Viability of ADM		
General Trust in ADM	0.67(0.03)	0.000
	*Intercepts*	
AK04.01	0.06(0.03)	0.032
AK04.02	– 0.12(0.03)	0.000
AK04.03	0.06(0.03)	0.064
VT08.01	– 0.04(0.03)	0.180
VT08.02	– 0.09(0.03)	0.002
VT08.03	0.04(0.03)	0.138
VT08.04	0.09(0.03)	0.003
	*Latent intercepts*	
Viability of ADM	1.04(0.08)	0.000
General Trust in ADM	2.76(0.02)	0.000
	*Latent variances*	
Viability of ADM	0.88(0.03)	0.000
General Trust in ADM	0.92(0.03)	0.000
	Fit Indices	
*χ*^2^	28.38(13)	0.008
RMSEA	0.03	
RMSEA.CI.LOWER	0.01	
RMSEA.CI.UPPER	0.04	
TLI	1.00	

The parameter estimate of the regression coefficient suggests a significant and strong effect of trust in AI on the perceived viability of ADM for vaccine distribution ($\beta$= 0.67, *SE* = 0.03, *p* < 0.01, $\beta$_standardized_ = 0.56). Accordingly, H1 is accepted.

*Relationship between disapproval of a social group’s vaccination prioritization and the legitimacy of early vaccination.* To test H2, we estimated a structural regression model. This model included the factorial survey condition as an independent variable using a dummy coded predictor (“vaccinate teachers first” = 0; “vaccinate prisoners first” = 1). The model shows good fit (see Table 6). The inadequate fit suggested by the RMSEA may be attributed to the model’s few degrees of freedom (Kenny et al. 2015).

**Table 6:** Structural regression model for the relationship between disapproval of a social group’s vaccination prioritization and the legitimacy of early vaccination

	Estimate (Std.Err.)	*p*
	*Factor loadings*	
Legitimacy of prioritization		
LG04.01	1.00(0.01)	0.000
LG04.02	1.05(0.01)	0.000
LG04.03	0.95(0.02)	0.000
LG04.04	1.01(0.01)	0.000
	*Regression slopes*	
Legitimacy of prioritization		
Disapproval of early vaccination	– 0.66(0.06)	0.000
	*Intercepts*	
LG04.01	0.151(0.03)	0.000
LG04.02	– 0.19(0.03)	0.000
LG04.03	– 0.06(0.04)	0.089
LG04.04	0.10(0.04)	0.000
	*Latent intercepts*	
Legitimacy of prioritization	3.34(0.04)	0.000
	*Latent variances*	
Legitimacy of prioritization	1.31(0.05)	0.000
	*Fit indices*	
*χ*^2^	174.47(23)	0.000
RMSEA	0.09	
RMSEA.CI.LOWER	0.08	
RMSEA.CI.UPPER	0.10	
TLI	0.98	

The parameter estimate of the regression coefficient suggests a significant medium negative effect by the factorial predictor on the perceived legitimacy of the decision ($\beta$ = − 0.66, *SE* = 0.06, *p* < 0.01, $\beta$_standardized_ = − 0.28). This means that the decision to first vaccinate a non-preferred group was judged as less legitimate than the decision to first vaccinate a group for which early vaccination was generally preferred. Accordingly, H2 is accepted.

*Moderation effect of trust in the agent making the decision* H3 assumes that trust in the agent making the decision will moderate the relationship between preference and decision legitimacy. More specifically, we expected that this negative relationship would be weaker when trust in the agent was high.

To test H3 and H4, we again utilized a structural regression model. This model included as independent variables the factorial survey condition as a dummy coded predictor (“vaccinate teachers first” = 0 versus “vaccinate prisoners first” = 1) and the trust in the agent making the decision. Additionally, a latent factor serving as the moderator variable was estimated based on indicators calculated as the products of the condition variable and the trust indicators using the *indProd*-function from the package *semTools* (Jorgensen et al. 2019).

The model shows good fit (see Table 7).

**Table 7:** Structural regression model for the moderation effect of trust in the agent making the decision

	Estimate(Std.Err.)	*p*
	*Regression slopes*	
Legitimacy of prioritization		
Trust in the agent	– 0.01(0.03)	0.728
Disapproval of early vaccination	– 0.66(0.06)	0.000
Disapproval of early vaccination * trust in the agent	0.02(0.05)	0.743
	*Latent intercepts*	
Legitimacy of prioritization	3.37(0.08)	0.000
Trust in the agent	2.85(0.03)	0.000
Disapproval of early vaccination * trust in the agent	– 0.00(0.01)	1.000
	*Latent variances*	
Legitimacy of prioritization	1.31(0.05)	0.000
Trust in the agent	1.35(0.05)	0.000
Disapproval of early vaccination * trust in the agent	0.34(0.01)	0.000
	*Latent covariances*	
Trust in the agent w/disapproval of early vaccination * trust in the agent	0.00(0.02)	1.000
	*Fit indices*	
*χ*^2^	160.97(60)	0.000
RMSEA	0.03	
RMSEA.CI.LOWER	0.03	
RMSEA.CI.UPPER	0.04	
TLI	0.99	

The parameter estimate of the moderator’s regression coefficient suggests no significant effect of the moderator variable on the perceived legitimacy of the decision ($\beta$= 0.02, *SE* = 0.05, *p* = 0.74, $\beta$_standardized_ = 0.01). In other words, trust in the agent making the decision had no moderating effect on the relationship between disapproval of early vaccination of a social group and perceived legitimacy of a decision for early vaccination. Accordingly, H3 is rejected.

*Moderation effect of the agent making the decision* H4 assumes a difference between a condition in which *either* ADM *or* HDM make decisions about early vaccination, in that the negative relationship between disapproval of vaccination and legitimacy of early vaccination is weaker for ADM making the decision when trust in ADM is high than for humans making the decision when trust in humans is high.

We estimated a structural regression model identical to the model estimated for H3. To assess the difference of the parameter estimates of the moderation, however, this model utilized multigroup analysis to compare the two groups in which *either* an ADM system decided vaccine prioritization *or* humans (i.e., the STIKO) did so.

The model shows good fit (see Table 8).

**Table 8:** Structural regression model for the moderation effect of the agent making the decision

	HDM		ADM	
	Estimate (Std.Err.)	p	Estimate (Std.Err.)	p
	*Regression slopes*			
Legitimacy of prioritization				
Trust in the agent	– 0.01(0.03)	0.649	– 0.01(0.03)	0.649
Disapproval of early vaccination	– 0.66(0.06)	0.000	– 0.66(0.06)	0.000
Disapproval of early vaccination * trust in the agent	0.07(0.07)	0.360	– 0.03(0.07)	0.653
	*Latent intercepts*			
Legitimacy of prioritization	3.39(0.09)	0.000	3.36(0.09)	0.000
Trust in the agent	2.93(0.04)	0.000	2.77(0.04)	0.000
Disapproval of early vaccination * trust in the agent	0.00(0.02)	0.962	– 0.00(0.02)	0.966
	*Latent variances*			
Legitimacy of prioritization	1.31(0.07)	0.000	1.31(0.07)	0.000
Trust in the agent	1.40(0.07)	0.000	1.28(0.07)	0.000
Disapproval of early vaccination * trust in the agent	0.35(0.02)	0.000	0.32(0.02)	0.000
	*Latent covariances*			
Trust in the agent w/disapproval of early vaccination * trust in the agent	0.00(0.03)	0.978	0.00(0.02)	0.996
	*Fit indices*			
χ^2^	316.58(141)	0.000		
RMSEA	0.04			
RMSEA.CI.LOWER	0.03			
RMSEA.CI.UPPER	0.05			
TLI	0.99			

Fixed parameter

The parameter estimate of the moderator’s regression coefficient showed no moderating effect of trust in the agent making the decision, either in the ADM condition ($\beta$ = – 0.03, *SE* = 0.07, *p* = 0.65, $\beta$_standardized_ = – 0.02) or in the HDM condition ($\beta$ = 0.07, *SE* = 0.07, *p* = 0.36, $\beta$_standardized_ = 0.03).

Furthermore, a test for parameter differences suggested that there was no significant difference between the moderating effects of trust in the two conditions ($\Delta \beta$= 0.01, *SE* = 0.10, *p* = 0.34, $\beta$_standardized_ = 0.05). H4 is also rejected.

## Discussion

In focusing on AI implementation to combat one of the biggest current challenges to humanity, namely COVID-19, our study adds to the research on a hotly debated social issue. As AI applications are already in extensive use that will most likely increase over the coming years, it is crucial to understand how the public perceives their widespread deployment, especially in high-risk situations. This study mainly focused on the role of trust and its effect on the perceived legitimacy of publicly preferred or unpreferred solutions.

The results of the factorial survey suggest that the German public is altogether indifferent about ADM usage to allocate vaccination against COVID-19. Answering our research question, the use of ADM to tackle this important issue is not rejected, but it also is not overly welcomed by German citizens. This insight is in line with research which suggests that, while German citizens are generally in favor of AI (bitkom 2018), they often show little interest in specific use cases (Meinungsmonitor Künstliche Intelligenz 2021). This raises questions about how to explain the public’s low interest in and involvement with ADM, especially in light of high expectations regarding the use of ADM in public administration (Wirtz and Müller 2018).

In confirming H1, we see that trust in ADM leads to greater acceptance of the use of ADM in the allocation of COVID-19 vaccines. This finding is also consistent with previous research, showing that trust positively affects the perceived satisfaction and usefulness of ADM systems (e.g., Shin 2020a; Shin and Park 2019). Hence, building trust in ADM systems will likely prove to be a fruitful way to generally legitimatize AI use in public administration decision-making. Consequently, it may be assumed that efforts to promote the use of ADM systems in the management of current crises would be widely accepted, especially by people who are generally in favor of AI and who show considerable trust in its beneficial potential. That being said, we have noted that trusting an agent does not necessarily mean that the agent is trustworthy. Regarding the real-world effects of ethical guidelines, Hagendorff (2020) argued that self-commitment to ethical criteria can function as strategic communication activity and does not necessarily imply factual adherence to those guidelines. Furthermore, Robinette et al. (2016) demonstrated the negative effects of overtrust in a high-stakes situation. Thus, further research should take into account actual differences in the trustworthiness between ADM systems of different designs in light of the nature of the respective high-stakes situation. As another limitation, we did not include general trust in other humans in our study; this could be included in future research that involves proposals of use of ADM systems. After all, mistrust of other humans may also help to explain a preference for ADM.

As initially well-received utilization may lead to unpopular and consequence-laden outcomes, we subsequently contrasted vaccine allocation decisions of high public preference with decisions of low public preference. Our findings reveal that ethical considerations might not be in line with—or might even strongly oppose—public preferences. For instance, prisoners are at high risk of contracting COVID-19 (Burki 2020). However, public sentiment strongly opposes the idea of prioritizing that group. This disapproval of early vaccination for an unpopular social group is negatively related to the perceived legitimacy of early vaccination for that group. These findings correspond to the literature on the allocation of scarce medical resources (e.g., Furnham et al. 2007, 2002; Ubel et al. 2001). Personal characteristics and life choices affect social preferences and influence how the public legitimates the prioritization of certain groups. Prisoners are assumedly being punished for a crime they committed, and the social preference for such persons is low in the German population, especially in contrast to teachers. Hence, public preference depends on the specific social characteristics that a group possesses (Luyten et al. 2020; Sprengholz et al. 2021). Existing studies on the allocation of scarce medical resources during the COVID-19 pandemic often do not differentiate based on the groups affected but rather on the ethical ground principles on which decisions are based (Huseynov et al. 2020; Grover et al. 2020). Thus, further studies should elaborate on our findings and probe into different preference patterns among the public to mitigate the detrimental effects of unpopular decisions on the general acceptance of ADM systems.

In a subsequent step, we asked whether trust moderates the link between social preferences and legitimacy. After all, trusting someone to make the right call may help one to accept an otherwise unpopular decision. Contrary to H3, in situations of significant discrepancy between expectations and actual outcomes, trust does not moderate the effect of social group preference on legitimacy. This suggests that trust is not the sole be-all and end-all when ADM is used to distribute public goods and that there are situations in which legitimacy does not depend on trust. Furthermore, there was no difference between ADM and HDM when it comes to this missing effect of trust. Thus, we do not find support in our data for either algorithmic appreciation (e.g., Logg et al. 2019) or algorithmic aversion (e.g., Dietvorst et al. 2015). In the high-stakes situation of COVID-19 vaccination allocation, it does not matter who decides. Hence, we could not replicate the effects reported by Araujo et al. (2020) or Robinette et al. (2016)—ADM systems were not especially preferred to human judgment in this high-impact situation. This finding has far-reaching implications. Based on the goal, algorithms are expected to produce accurate and objective results. On one hand, ADM systems are supposed to arrive at ethically sound decisions (e.g., as required by the high-level experts of the 2019 European Commission). On the other hand, correct and ethically tenable outcomes may not be in line with the opinions of the general public. As the overarching goal is to build trustworthy AI systems that benefit the Common Good, this points to a potentially major conflict, as not all of these demands may be satisfactorily met at all times. Hence, we show that building trustworthy and ethically sound ADM systems may not be the solution to every ethical problem in the eye of the public. As ADM is increasingly integrated into society, it is crucial to keep these findings in mind. We are far distant from the point at which the general public will wholeheartedly trust the decisions of a machine. Legitimacy is first and foremost influenced, at least in our case, by public preferences related to the solution an agent proposes.

## Implications

Our study has implications for both academic research and the practical use of ADM systems. For citizens, in the present scenario, it matters whether a decision concerning the allocation of vaccines follows their personal preferences, irrespective of the agent who makes the decision and their trust in that agent. Adding to the literature on algorithmic trust, our study suggests that trusting an ADM generally leads to higher perceived legitimacy. However, regarding studies that compare trust in humans and trust in ADM, we find support for neither algorithmic aversion nor algorithmic appreciation of the decisions in our case example. When taking the context of high-impact decision-making into account, we cannot replicate empirical findings that document a preference for ADM systems over humans. This might be explained by some of the limitations of our study. First, our study was conducted among German citizens, who are comparatively critical towards ADM systems. Second, unlike Araujo et al. (2020), we did not compare a high-impact decision with a low-impact decision. On the other hand, in spite of research on trust and ADM that expects conciliatory effects of trust and the nature of the agent on the general legitimacy of decision-making, there seem to be limits to the prevalence and strength of those presumed effects. Future research should disentangle these findings and identify critical inflection points where, as suggested in the literature, moderating effects may still be found (e.g., Shin and Park 2019). Thus, further studies should elaborate the situational and cultural contexts of different high-impact decisions and the connection to trust in specific (un-)trustworthy ADM systems.

Regarding ethical guidelines and striving toward trustworthy ADM systems, we conclude the following: While we strongly support ethical AI guidelines, we observe that ADM decisions and demands for trustworthy AI may sometimes not be in line (and in fact may be in direct conflict) with public perceptions of AI’s output. Thus, alongside the development of ethical AI in technical terms, companies and researchers must also acknowledge the relevance of public opinion. As seen in the case of vaccine distribution in the USA (Guo and Hao 2020) and Germany (Ciesielski et al. 2021), which often created misleading, unexpected, and unpopular results, particular outcomes may backfire and fuel public outrage against the use of ADM. Hence, decision-makers must weigh ethical considerations and the public’s will in light of probable public resistance to ADM decisions.

Regarding the impact of ethical guidelines on subsequent research, these findings could lead into two directions. First, political decision-makers may be even more reluctant to set binding regulations for ethical ADM development, as these may oppose public opinion, which they need to take into account as it is important to legitimize their power. When the use of ADM systems leads to decisions—whether in accordance with specific norms and ethics or not—that are in conflict with the social preferences of citizens, political actors risk public scorn. Thus, following the implementation of strict criteria may not be in the interest of decision-makers, and future research may shed light on the consequences this may have for political behavior. Second, if issues of trustworthiness and social preferences arise, decision-makers must be prepared to engage with the public to mitigate potential conflicts by, for instance, more thoroughly explaining the ethical dilemma and justifying unpopular decisions. As a potential remedy to this dilemma, studies focusing on *Explainable AI* (XAI) highlight the importance of explaining ADM’s forecasts and the resulting decisions to citizens (for an overview, see Miller 2019). Empirical studies have found that explaining ADM decisions leads to greater trust in those systems and, in turn, to greater acceptance (Shin 2021b). Thus, further studies could enhance our design—in which decisions regarding vaccine allocation were neither explained nor justified—and test if a more or less detailed and comprehensible explanation for a decisive outcome would soften the negative effect of social group preference on perceived decision legitimacy. After all, the conflict between ethical decisions and their negative public perception in light of public opinion may be mitigated with specific communicative strategies involving convincing explanations that make the inner workings of ADM comprehensible to a lay audience.

On a practical note, decision-makers, be they politicians, administrators, or developers, have to carefully weigh the risks and benefits of using ADM systems for COVID-19 vaccine allocation and for other cases where public goods need to be distributed. While those systems may certainly have positive effects, such as speeding up logistical processes, ADM systems must be carefully evaluated before their implementation. The first step should be an evaluation of their ethical soundness and possible negative consequences (e.g., their discrimination potential). Second, to preempt potential conflict, decision-makers need to be transparent and proactive about the use of ADM systems, their inner workings, and the possible consequences of their distribution of public goods. Thus, it might be useful to research and develop accompanying communication strategies for engaging the public that also take into account conflicting public perceptions and interests. To mitigate potential public fallout related to social issues hitherto not appropriately considered in the use of ADM, such communicative initiatives for public engagement may help raise greater awareness of the use and limitations of ADM and may thus begin to reconcile the existing social dilemma within the public sphere.

## Conclusion

The vaccination program against the novel coronavirus currently poses a challenge of global dimensions and, as such, is the subject of a controversial social debate. Decision-makers have to allocate scarce medical resources while considering many factors, including practical and moral questions as well as consideration of public opinion. ADM systems can be deployed to support this process by providing suggestions or even autonomously deciding upon the priority order for vaccination.

Our research suggests that the use of ADM to combat COVID-19 is only ambivalently perceived to be a viable strategy by the German public and that general trust in AI is an essential driver of viability perceptions. However, irrespective of actual discrimination—be it necessary or faulty—by ADM, we show that proposal of publicly unpreferred decisions regarding the allocation of vaccines leads to these decisions being perceived as less legitimate. We subsequently inquired about the moderating role of trust in the agents making decisions on the legitimacy of unpreferred decisions in the allocation process. Contrary to expectations, trust in the agent did not have the expected mitigating effect. As there was also no difference between HDM and ADM, this raises important questions for researchers and decision-makers concerning the expected future deployments of ADM for administrative decision-making. As there are potentially many ethically correct and preferable yet widely unpopular decisions that ADM systems will propose in the future, we conclude that there are severe challenges for current initiatives that promote the implementation of trustworthy AI.

## Availability of data, code, and materials

The data and code for data analysis used in this study can be accessed via the project’s Open Science Foundation repository (Link: https://osf.io/xhvwr).

## Footnotes

1 In the following, we report on two examples of the use of ADM in COVID-19 vaccine allocation, one from the USA and one from Germany. We’ve chosen these examples, because they were—to our knowledge—the first practical utilization of ADM for vaccine allocation. Additionally, both cases gained some media attention. However, there are other examples of the use of ADM to allocate COVID-19 vaccinations that were implemented later in 2021, including in the UK (Philpotts 2021) and in Italy (Chiusi 2021).

2 We note that we do not research the interaction of the fulfillment of ethical principles to achieve trustworthy AI in this paper. However, a recent study by Kieslich et al. (2021a) explores the relative importance of these principles in the public’s eye. In this paper, we solely focus on the effects of trust perceptions of ADM compared with HDM.

3 As discussed above, ADM systems may be regarded as a form of AI. Consequently, some questions used in the questionnaire utilize the terminology of AI. On one hand, it arguably is a more familiar term for the German public than is ADM. On the other hand, we aimed to measure attitudes about the technology on a broader level.

4 We utilized the quotas provided by respondi, which they regularly use in their surveys. The quotas for age and gender were drawn from Eurostat and the quota for educational level from the 2019 b4p report. Quotas are as follows: gender (female = 49.75%; male = 50.25%), age (18–29 years = 19.40%; 30–39 years = 17.46%; 40–49 years = 17.93%; 50–59 years = 22.33%; 60–74 years = 22.88%), and educational level (basic educational attainment = 33%; medium educational attainment = 32%; advanced degree = 35%).

5 The other groups were personnel in health care professions, police and firefighters, employees in the meat processing industry, persons belonging to a medical risk group, children and adolescents, young people who frequently travel in groups, people of retirement age, and students.

6 The variable IDs for trust in ADM for vaccine distribution are VT06_01, VT06_02, VT06_03, and VT06_04. The variable IDs for trust in HDM for vaccine distribution are VT07_01, VT07_02, VT07_03, and VT07_04. As respondents either received the ADM or HDM scenario, both variables were merged into one variable named moderator1, moderator2, moderator3, or moderator4 for the data analysis of H4.

7 Sociodemographic variables have not been included in the models, as “control variables should only be included when theoretically relevant” (Bol et al. 2018, 16). There were no theory-based assumptions regarding the influence of such variables on the distribution of public goods using ADM. For instance, Araujo et al. (2020) report no effects for the case most similar to our case (i.e., fairness perceptions). We thus were primarily concerned with answering our research question and testing our hypotheses, and we focus on the theorized effect itself in our population rather than on its prevalence and strength within subpopulations.

8 Following Hu and Bentler (1999), we follow their recommendation of a value of TLI $\ge$ 0.95 and RMSEA $\ge$ 0.06 for assessing the goodness of fit of the estimated models.
